# Advances in Tools to Determine the Glycan-Binding Specificities of Lectins and Antibodies*

**DOI:** 10.1074/mcp.R119.001836

**Published:** 2019-12-17

**Authors:** Brian B. Haab, Zachary Klamer

**Affiliations:** Van Andel Institute, Grand Rapids, Michigan

**Keywords:** Glycoproteins, glycosylation, bioinformatics, glycomics, micro arrays, glycan arrays, lectins

## Abstract

Proteins that bind carbohydrate structures can serve as tools to quantify or localize specific glycans in biological specimens. Such proteins, including lectins and glycan-binding antibodies, are particularly valuable if accurate information is available about the glycans that a protein binds. Glycan arrays have been transformational for uncovering rich information about the nuances and complexities of glycan-binding specificity. A challenge, however, has been the analysis of the data. Because protein-glycan interactions are so complex, simplistic modes of analyzing the data and describing glycan-binding specificities have proven inadequate in many cases. This review surveys the methods for handling high-content data on protein-glycan interactions. We contrast the approaches that have been demonstrated and provide an overview of the resources that are available. We also give an outlook on the promising experimental technologies for generating new insights into protein-glycan interactions, as well as a perspective on the limitations that currently face the field.

## Abstract (precis)

Lectins and glycan-binding antibodies are powerful tools in biological research, provided detailed information is available about their glycan-binding specificities. Glycan-arrays, in combination with bioinformatics tools to mine the data, offer the ability to obtain such information. This review focuses on the bioinformatics tools and resources that are available for the analysis of glycan-array data. The tools are enabling new insights into protein-glycan interactions and enhancing the value of glycan-binding proteins in research.

## Graphical Abstract

**Figure:**
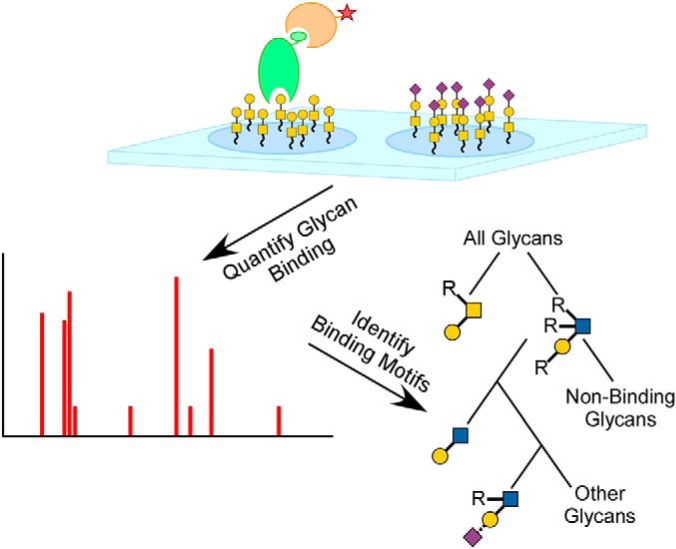

**Highlights**

- Lectins and glycan-binding antibodies are valuable as probe of glycans.
- Advanced bioinformatics tools enable the mining of glycan-array data.
- New insights into protein-glycan interactions have value in biological research.

Glycans are a fundamental part of biology. They cover cell surfaces, decorate most secreted proteins, control access to cells, and modify protein-protein and inter-cellular interactions. Glycans form the first-line mode of communication between the microbial world and the human, animal, and plant systems, and they form a main component of innate immune recognition. The adaptive immune system also relies heavily on glycan recognition, contrary to previous predictions, as shown by the large percentage of antibodies in the circulation that recognize glycan epitopes (1). Thus, researchers from diverse fields find glycans a fascinating topic of study.

A common feature among the many fields of study is this: obtaining information about glycan functions and structures is challenging. Researchers do not have the types of well-developed tools that are available for studies of nucleic acids and proteins. Automated synthesis of glycans structures, or the amplification of sequences using biotechnology, are not available. Sequencers to conveniently determine the monosaccharide backbone and linkages of a glycan are not available. And methods to induce or knock-out glycan structures on a specific protein are not available. The tools that are available are the domain of specialists, for the most part, excepting the basic methods to obtain rudimentary information. This situation is improving, owing to efforts on the part of funding agencies and tool developers to bring accessible tools to researchers, but gradually.

In this review, we focus on an approach that has long-standing use in the biological research community and that has the potential for increased and broader value. This approach is the use of affinity reagents, or glycan-binding proteins. Glycan-binding proteins, which include native proteins that bind specific glycan structures, termed lectins, and antibodies that recognize glycans, are used in the same way that antibodies are widely used to study proteins. They can be used to quantify specific features in biological samples or to identify the locations of the features in tissue or on cell surfaces, for example. Lectins have been used in this way for decades (2). The advantages of using lectins and antibodies for studying glycans is that they are easy to use in many types of experiments, they are inexpensive, the assays can be quantitative and high-throughput, and they give measurements about specific glycan motifs or features.

Owing to these advantages, lectins remain the primary method for identifying and quantifying glycan structures in biological samples or on proteins. But this approach also has limitations. The experiments do not give information about complete monosaccharide compositions, or about the heterogeneity of glycosylation between or within proteins, or about the locations of glycosylation on protein backbones. Such information can be accessed through increasingly sophisticated mass spectrometry methods.

Nevertheless, researchers are advancing the use of lectins in biological research. One of the most important advances is improved information about the binding-specificities of lectins. This progress results from improvements in both the experimental methods and the bioinformatics tools. Here we focus primarily on the bioinformatics tools that enhance the value of the experimental data. Developments in the experimental methods are too numerous to be covered in this review. For the researcher who is not a developer of technology, we provide an overview of the experimental and data resources that are currently available and a broad survey of experimental innovations that could eventually provide value to the research community.

## 

### 

#### Available Experimental and Data Resources

The driving technology in the study of lectin-glycan interactions has been the glycan array. Prior to the introduction of glycan arrays in 2002 (7–9), studies of lectin-glycan binding interactions required serial analyses of individual interactions, for example using elutions from affinity gels or competitive inhibitions of binding (3). These methods required large amounts of each glycan and had limited ability to test many interactions. Subsequent methods providing increased throughput and precision include frontal affinity chromatography (4) and surface-plasmon resonance (5, 6). But the glycan array opened the possibility of probing in parallel dozens of glycans, using tiny amounts of each glycan (Fig. 1).

**Fig. 1.**
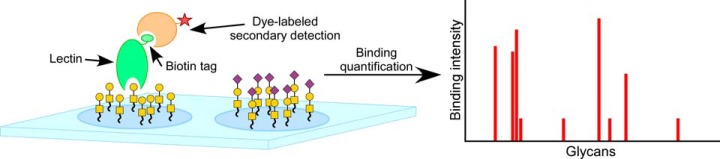
**The acquisition of glycan-array data.** The typical experiment involves incubating a lectin or glycan-binding antibody on a microarray of diverse glycans, followed by quantifying the amount of binding to each glycan. The protein usually is labeled with a fluorescent tag or another tag that allows fluorescence detection by a secondary agent.

Researchers have produced a huge amount of glycan-array data since the introduction of the technology. The glycan arrays produced by the Consortium for Functional Glycomics (CFG)—a project funded by the National Institutes of Health—have been particularly popular (10), and the data and have been extensively accessed. The public funding was valuable for initiating developments, but several companies are now providing longer-term options for access to glycan-analysis. Core services in academic settings also are offering options as the methods become further standardized. Table I provides a summary of the academic and commercial arrays and datasets that are available as resources. The Table is not exhaustive list of arrays produced by any group, but rather includes those that are provided as a general service; additional arrays produced by academic groups are referenced in the Developments in the Experimental Methods section. In addition, data from glycan arrays other than the CFG array are available through the supplementary data corresponding to publications.

**Table I:** Experimental and data resources for glycan arrays. The criterion for inclusion was any array that advertised itself as a service or resource and that had appropriate web-accessible information and request forms

Source Type	Source	Content Type	Number of Glycans	Data Available
Academic	National Center for Functional Glycomics (NCFG)	Previous Versions of the CFG	250–600+	Yes
		Current Version of the CFG	600	Yes
		Mannose-6P Array	26	No
		Modified Sialic Acid Array	80	No
		NCFG General Glycan Array	100	No
		NCFG SBA Array	106	No
		Asparagine-Linked Array	38	Yes
	Imperial College	Custom Arrays	796	Yes
Commercial	Z Biotech	General Array	100	No
		N-Glycan Array	100	No
		O-Glycan Array	94	No
		Heparan Sulfate	24	No
		Neu5Gc/Neu5Ac	80	No
		Human Milk Oligosaccharide	46	No
		Glycosphingolipid	58	No
		Glycosaminoglycan	34	No
	Chemily	Blood Group Antigen	21	No
		General Array	100	No
		General Array	300	No
		Glycosphingolipid	58	No
		Human Milk Oligosaccharide	46	No
		N-Glycan Array	100	No
		Neu5Gc/Neu5Ac	80	No
	RayBiotech	General Array	100	No
		General Array	300	No

#### Software for Determining Glycan-Binding Specificities

The analysis of glycan-array data has the goal of uncovering the rules that govern the binding of a protein to glycans. One can ask the question, what are the features of a glycan that determine whether a lectin binds or does not bind, or that tune the level of binding? Sometimes the rules appear relatively straightforward. In the case of *Vicea villosa* lectin (VVL), the presence of a terminal, alpha-linked N-acetyl-galactosamine is necessary and enough for binding to all glycans tested so far. For the complex cases, the rules may involve longer-range interactions with neighboring monosaccharides or separate branches (11, 12). Recent studies of human intelectin-1 (13) and DC-SIGN (14) provide examples of complex rules governing glycan recognition.

Visual inspection of the data can provide qualitative assessments of glycan-binding specificity. This system can function sufficiently well in many cases, such as in studies of changes in influenza specificities (15, 16). But manual analyses have disadvantages. They require expert knowledge; they are subject to the bias of the interpreter; the specificities of proteins are often too complex to be accurately discerned by visual inspection and described by qualitative terms; and they are not amenable to high-throughput processing. Therefore, algorithms for computer analyses are necessary.

To develop an algorithm for glycan-array analysis, one needs a method of describing the potential binding-determinants of a protein, or the glycan motifs. The glycan motifs are the substructures or patterns of monosaccharides that potentially are bound by a lectin (Fig. 2*A*). A method of describing motifs enables glycan-array analyses using the basic approach of (1) determining the presence or absence of the motifs on the glycans of an array, and (2) identifying the relationships between the motifs and the binding of the lectin. This approach was demonstrated by Porter and coworkers in 2010 (17). It is analogous to identifying the DNA motifs bound by a transcription factor, but with added complexity. A lectin does not bind a static substructure, but rather a family of substructures, some members stronger and others weaker. The contact points between a lectin and a glycan could involve monosaccharides that are non-contiguous or on different branches. Thus, some monosaccharides could be interchangeable, and the distances between contacts could be variable. Developing a notation to define the glycan motifs that accurately portray lectin binding has been the ongoing challenge for glycan-array analyses (Fig. 2*A*).

**Fig. 2.**
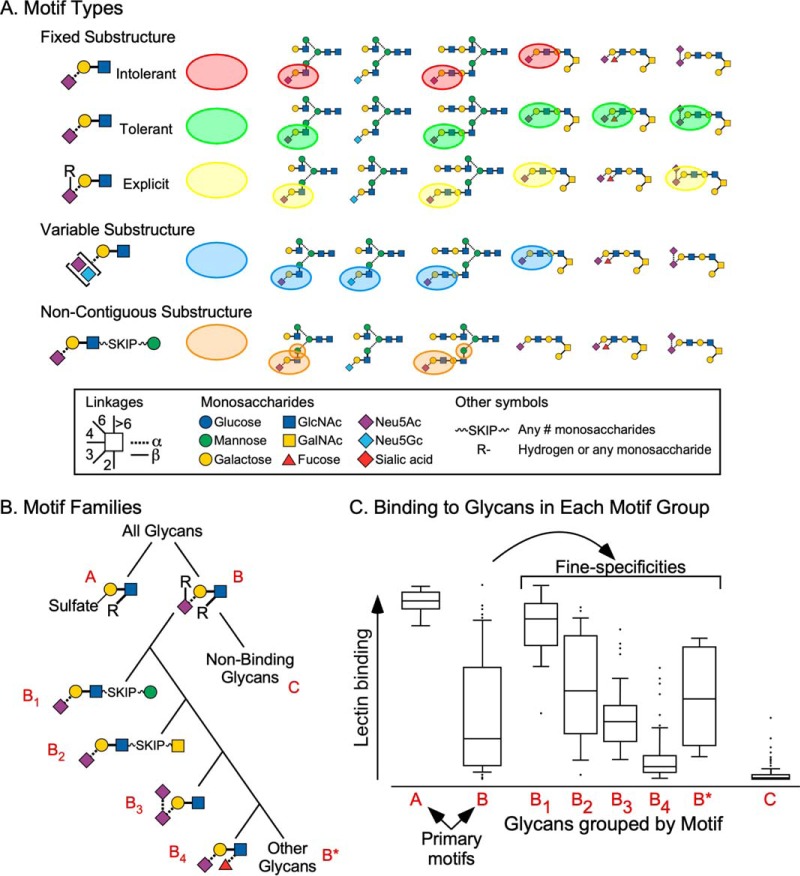
**Defining motifs and families of motifs.** *A*, Motif types. Fixed substructures are continuous units of defined monosaccharides. Intolerant definitions require the unit to be unsubstituted, and tolerant definitions allow substitutions. Explicit definitions define the locations where substitutions are optional, which gives the highest level of precision in the definition. Variable substructures allow for options in the monosaccharides, providing another level of flexibility in the definition. Non-contiguous substructures allow the components of a motif to be physically separated. This feature is useful when a lectin contacts separate branches of a glycan. *B*, Motif families. The tree shows the relationships between the groups of glycans with the indicated motifs, using a simulated analysis. The first split represents primary motifs (A and B) to which a protein binds. Motif B can be split into sub-motifs that represent fine specificities. *C*, The simulated data show the ranges of lectin binding to the glycans in each of the motif groups. For example, the glycans in group B contain motif B but not motif A. The B_1_-B_4_ sub-motifs define fine-specificities with differing ranges of binding, potentially explaining the broad range of the parent motif B.

The Porter work used motif definitions based on patterns that are common in mammalian biology. This method had the advantage of incorporating expert knowledge, and it proved accurate in identifying the main specificities of 90 different lectins using data in the CFG database. The user can add new motif definitions, based on additional analyses, to more-accurately describe binding (18). Automated processing of glycan-array data provided global analyses of over 3000 datasets in the CFG database (19).

Another system had the goal of computer-based motif discovery (20), as opposed to user-defined motifs, based on the rationale that an algorithm could pick out unusual specificities that might be missed by a user. The GlycanMotifMiner algorithm identifies the glycans with high binding and uses an iterative search for a subtree that is enriched in the high-binding glycans. The method tests monosaccharide additions to a starting monosaccharide, and then grows the subtree until any addition results in too few binders or too many non-binders. This method has the advantage of not requiring pre-defined or user-refined motifs, but it also has limited ability to find complex specificities, owing to the use of contiguous subtrees as motifs, which do not allow substitutions or gaps between monosaccharides and other complexities. It also requires dichotomizing the glycans into binders and non-binders, which is not a clear distinction in many datasets. A web platform provided convenient access to the GlycoPattern program, which serviced the CFG-array data (21). Related methods have been developed with variations including the use of kernel methods (22) and the use of alpha-closed subtrees (23, 24). These methods showed value in the identification of non-sialylated motifs bound by the influenza virus (25).

A method that incorporates more flexibility into the motifs was demonstrated by Hosada *et al.* (26). The Multiple Carbohydrate Alignment with Weights (MCAW) algorithm adapted a sequence-alignment algorithm commonly used for DNA alignments, called ClustalW (27). The method aligns the glycans that are strongly-bound by a protein to find a consensus sequence. The consensus sequences are scored by the similarity of the monomers and penalized for gaps. The authors demonstrated the method's effectiveness by analyzing over 1000 CFG data sets and distributing the results in a web-accessible database (28). The method can identify the locations where variability is allowed or disallowed, but disadvantages are that it does not narrow in on the minimal features required for binding, and that it provides little information on lower-affinity motifs. The method could provide interesting insights that are not apparent from other methods, however, and it demonstrates a novel adaptation of DNA-oriented bioinformatics for glyco-bioinformatics.

We previously introduced a method that has the potential of accounting for the complexities of lectin and antibody binding. The method is built on two primary features: (1) flexibility in individual motifs; and (2) families of motifs. The first feature accounts for the variability in the binding-site of a protein. We developed a new syntax, or motif language (29), that uses wildcards and logical operators (AND, OR, etc.) to describe variability in monosaccharides or linkages, and that uses other modifiers to allow for gaps of any length. The monosaccharide carbons can be defined either as “free” (cannot be substituted) or as tolerating substitution, which then distinguishes a terminating monosaccharide from an internal monosaccharide. The result is that motifs of nearly any variability or complexity can be represented (Fig. 2*A*).

The second feature, using families of motifs (Fig. 2*B*), accounts for the fact that not all the allowed binding partners of a protein are alike; some modifications to a glycan tune the binding to make it stronger or weaker. These sub-motifs are the fine specificities of a glycan-binding protein. In addition, proteins can have alternate specificities. Unlike fine specificities, primary specificities are different motifs altogether. For example, Concanavalin A primarily binds mannose as found in *N*-linked glycans, but it also binds terminal, alpha-linked glucose. Another example is given by wheat-germ agglutinin (WGA)^1^, which has separate preferences for N-acetyl-lactosamine, terminal GlcNAc or GalNAc, or sialic acid. We therefore represent protein binding not as a single motif, but as a family of motifs, with the relationships between motifs organized as primary motifs and fine specificities. The relationships can be visualized graphically (Fig. 2*B*).

These features are the foundations of the MotifFinder software (29) for automated analyses of glycan-array data. The program searches for the individual motifs that best describe specific fine-specificities or primary-specificities, and it searches for the set of motifs and the relationships between them that account for the overall binding pattern of the protein (Fig. 2*C*). The flexible motif syntax enables the automated generation of novel motif definitions that can account for unforeseen fine-specificities. This capability could be useful for characterizing protein binding to unusual glycans, as in a previous application of the method (29) to glycans with unequal extensions and substituents on each branch (30). The method identified features that would not have been practically analyzable using manual analyses or previous modes of representing motifs.

#### Applying the Information

One benefit of the high-precision characterization of glycan-binding specificity is insight into the biology of protein-glycan interactions. Another benefit is the improved use of lectins and antibodies to analyze glycans in biological material. For example, instead of using manual analysis to give a simple, qualitative interpretation of the presence of a motif, a software tool could provide quantitative estimates of glycan motifs. This approach could account for complex and nuanced aspects of protein specificity, and it opens the possibility of integrating information from multiple proteins. A researcher could probe a sample with multiple, different lectins, and the software could use the profile of binding levels across the lectins, combined with the detailed determinations of motif preferences from glycan-array analyses, to provide estimates of the amounts of multiple motifs (Fig. 3). Such datasets are frequently collected using lectin arrays (31, 32).

**Fig. 3.**
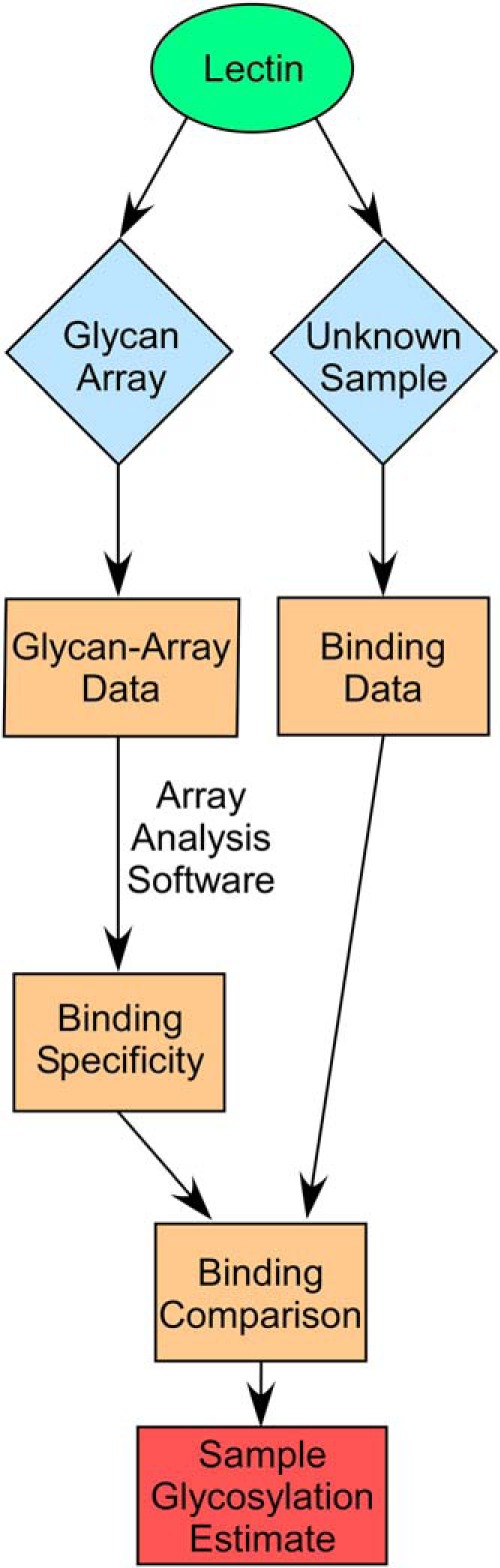
**Using the results from glycan-array analyses to interpret experimental data.** A lectin (or a glycan-binding antibody) can be applied to glycan arrays and experimental samples in separate experiments. Analysis software is applied to the glycan-array data to determine the lectin specificities, and the amount of lectin binding to the sample with unknown glycans is quantified. The output from the glycan-array analysis is combined with the data from the sample to produce an estimation of the glycans that are present in the sample. This scheme also could be used with integrated data from multiple lectins, and with data acquired after treatments with glycosidases.

Further, one could apply lectin profiling after rounds of exoglycosidase cleavage in order to obtain additional information about the structures. Lectin probing in the presence or absence of glycosidase digestion has been used in affinity-electrophoresis, lectin-blotting, and histochemical analysis (33–36), and we have extended the approach to a micro-scale format in combination with algorithms for the automated interpretation of the data (37, 38). We proposed that this method could provide complementary information to mass-spectrometry and be practical for researchers who are not experts in glycobiology.

#### Available Software Resources

Table II provides an overview of software resources that are available for glycan-array analysis.

**Table II:** Software resources for glycan array analyses

Software	Method	Summary	Reference
MotifFinder	Motif Binding Association	Uses multiple statistics to test the associations of motifs with lectin binding, using an advanced motif syntax that allows flexible motif definitions. Recent versions include automated motif optimization and modeling families of motifs.	Klamer 2017 (29)
GlycoPattern	Frequent Subtree Mining	Uses a graph theory approach to mine new glycan substructures that are frequent in the bound glycans and infrequent in the unbound glycans.	Cholleti 2012 (20) Agravat 2014 (21)
Multiple Carbohydrate Alignment with Weights (MCAW)	Weighted Structure Alignment	Adapts traditional sequence alignment algorithms to align the strongest glycan binders for a lectin. Offers a database of analyzed CFG datasets.	Hosoda 2017 (26) Hosoda 2018 (28)
GLycan Array Dashboard (GLAD)	Graphical Visualization	Enables researchers to explore trends in glycan array data through graphic visualization and the manual exploration of simple motifs.	Mehta 2019[1]

#### Developments in the Experimental Methods

Recent publications (39–43) give good reviews of the many developments in glycan array technology. The approaches differ in fundamental areas such as the production of the glycans, the presentation of the glycans, and the quantification of lectin-glycan interactions. In the end, no single platform gives a complete picture. No platform has all the glycans necessary for such a picture, and each platform has constraints that could influence binding patterns. Direct comparisons between platforms showed that the results can be divergent (44, 45). Many groups have pursued sophisticated enhancements to the experimental systems, and the field has grown beyond what can be reviewed here. Below we provide a sampling of the important work that eventually could be useful for researchers in biology.

A theme that has engaged many technology-developers is the better modeling of the biological environment. Most lectins occur as multimers of repeating subunits (46, 47). For example, the *Aurelia aurantia* lectin (AAL) has a 6-fold beta-propeller structure with five fucose binding sites on the edges (48, 49). The repeated glycan-binding sites are thought to increase avidity to glycans presented in corresponding units on cell surfaces, where they can change densities (50, 51), or in closely spaced arrangements on a protein. The experimental investigation of this effect using conventional arrays is limited. To produce glycans facilitate studies of multivalency, researchers have synthesized glycopolymers or glycodendrimers, in which glycans decorate a polymer backbone at controlled intervals (52–55). This method offers unique insights into the avidities of hetero-multivalent binding, although it is limited in breadth by the significant synthetic hurdle. Another approach is to measure the agglutination of emulsions containing mixtures of two glycans (56), which enables studies of the kinetics of hetero-multivalent binding and more accurately models a membrane environment. Glycans attached to quantum dots (57) also could be useful for studying multivalent binding, because the glycans can be kept in proximity in the solution phase. An approach that is easier to implement and higher-throughput is to vary the numbers of glycans attached to a protein carrier (58). This method can reveal density-dependent effects but has less control over the molecular details. Bead-based formats (59, 60) could give increased flexibility in experimental design and solution-phase interactions that are not available using planar arrays.

Label-free methods also could provide improved measurements of certain glycan-protein interactions, because the chemical labeling of a protein could affect binding. Surface plasmon resonance offers measurements of binding affinities as well as the ability to identify low-affinity interactions (61, 62). Mass spectrometry could provide solution-phase detection of the glycans bound by a protein and potentially more accurate measurements of binding strengths relative to solid-phase methods (63). A demonstration of this approach using catch-and-release system allowed the assay of glycans bound by various glycan-binding proteins (64, 65). A related method utilized a universal proxy-protein receptor to allow the quantitative screening of glycan binding and carbohydrate-active enzyme activity (66). An inherent challenge with mass-spectrometry is distinguishing between glycans that have the same mass but differences in linkage or sequence, which occur frequently and are functionally important.

Expanding the range of glycans available for the experiments is another significant goal. In contrast to proteins and nucleic acids, glycans need to be synthesized individually or purified from natural sources. In addition, they cannot be amplified through biotechnology. Purification from natural sources is an attractive option for glycans that are not amenable to synthesis and that directly relate to biological samples. The purified glycans could be attached to a linker (67, 68) or fluorescent tag (69, 70) for further analysis. Disadvantages are the difficulty in achieving full purity and the need for structural characterization after purification. Synthetic strategies address these limitations and are making good progress. New synthetic methods have provided structures that previously were difficult to synthesize, such as asymmetrically-branched N-glycans (29, 71–73), which were useful for producing arrays of human milk oligosaccharides (30). Several groups have developed arrays for additional classes such as sialylated structures (74–76), plant cell-wall glycans (77), and microbial glycans (8, 78, 79). The synthesis of these structures currently is limited by the requirement for high expertise and customization, but automated synthesis, which was shown to be feasible selected glycans using enzyme-mediated methods (80, 81), could alleviate that bottleneck.

Though tangential to the *in vitro* methods, it is worth noting the complementarity of structural analysis and simulation. Grant *et al.* used computational grafting to generate putative structures for lectin-glycan complexes, giving justification for the observed patterns of binding on a glycan array (12). Anomalous experimental data was explained by the hindered access of bulky, multi-subunit lectins to glycans that are too close to the surface (82). Sood *et al.* used molecular-dynamics simulations to quantify the contributions made by the functional groups of monosaccharides and to identify the minimum features required for lectin-glycan binding (83). A tool that could facilitate these types of analyses is a curated database called Unilectin3D, which lists the structures in the Protein Database (PDB) of lectins in complex with a glycan (84). The in-silico methods could provide valuable context to the experimental results and produce accurate predictions of binding to glycans that are not represented on the arrays.

#### Outlook

The resources and tools available to researchers have greatly increased in recent years, with more in development. In addition to the developments covered here, mass-spectrometry methods for glycan analysis are advancing in capabilities and availability, and molecular biology methods involving genetic and chemical manipulations are increasingly powerful. Researchers will generally need to draw upon several approaches to thoroughly study questions. Thus, each of the methods need to be standardized and accessible to researchers who are not specialists in the technologies. In addition, software will be needed to integrate information from multiple, disparate sources.

For glycan-array methods and analyses, researchers would benefit from the calibration of the experiments through standardized material. Well-characterized glycoproteins or manufactured glycoproteins could be used to calibrate data on quantitative scales. Standards also could provide a means to link information between experiments. The published guidelines for glycomics experiments (85) are helpful but do not address standards for calibration. Another need is data repositories. Many groups are producing data that could be useful for others, especially from glycan-array and mass-spectrometry experiments, but databases for deposition are not available. The data are either not available or are spread across the supplementary data of hundreds of papers. In the analogous domains of protein and nucleic-acid research, several databases are accessible for the raw or processed data, such as GEO for gene expression, PDB for structural biology, and many more. Although some glycan-binding datasets have been submitted to the GEO database, this repository is ill-suited for glycan-binding data and is not an intuitive location to find such data.

Another major need is improved information about glycosidase specificity. Glycosidases are standard tools in glycan sequencing by chromatography and electrophoresis, and they are increasingly used in mass-spectrometry experiments. They also can be used to uncover motifs to be probed by lectins (37, 38, 86). In many cases, only basic information is known about the specificity of a glycosidase. Glycan arrays could help by enabling measurements of activity over a huge number of glycans, as demonstrated in a study of influenza neuraminidase (87). Software to interpret data from glycan arrays that were treated by glycosidases and then probed by lectins (38) could provide detailed insights into glycosidase specificity and a means to apply the information.

## Footnotes

^1^ The abbreviations used are:

WGA: wheat-germ agglutinin
PDB: protein database.
